# Development and application of a TaqMan probe-based pentaplex qPCR for the rapid detection and differential identification of five pathogens

**DOI:** 10.3389/fmicb.2026.1841879

**Published:** 2026-06-02

**Authors:** Zheng-qin Gao, Rui Fu, Jin Xing, Shan-shan Yao

**Affiliations:** National Institutes for Food and Drug Control, Beijing, China

**Keywords:** differential identification, pathogens, pentaplex qPCR, rapid detection, TaqMan probe

## Abstract

*Cryptosporidium parvum* (Cp), *Strongyloides stercoralis* (Ss), *Trypanosoma cruzi* (Tc), *Theileria ovis* (To) and *Echinococcus granulosus* (Eg) are significant in causing similar clinical symptoms in both humans and animals. These symptoms include diarrhea, fever, pneumonia, hepatitis, encephalitis, cardiovascular, and gastrointestinal diseases, as well as reproductive system diseases. Therefore, it is urgent to establish a rapid, specific, and sensitive method to simultaneously detect these five pathogens. Here, we develop a TaqMan probe-based pentaplex real-time fluorescence quantitative PCR (qPCR) for simultaneous detection of Cp, Ss, Tc, To, and Eg for the first time. Five sets of specific primers and probes were designed. The 5′ end of five probes were labeled with five different fluorescent groups (FAM, VIC, ABY, JUN, CY5) that do not interfere with each other, while the 3′ end were labeled with quenching groups (MGB-NFQ, QSY). The instrument collects fluorescence signals at different wavelengths in five independent optical channels in qPCR to achieve simultaneous specific amplification and quantitative detection of five targets within one tube. A five-color qPCR was developed, which can rapidly and quantitatively distinguish and identify Cp, Ss, Tc, To, and Eg in a single tube. This method shows strong specificity and no cross-reaction with other control pathogens. This method has high sensitivity, with the LLOQ of 10 copies and the LOD as low as 1 copy. The standard curve shows a good linear relationship. The amplification efficiency is high. The repeatability and reproducibility are excellent. The interference test shows that high concentrations of nucleic acids do not interfere with low concentrations of nucleic acids, and can ensure excellent detection results even in complex samples for 1.5 hours. A total of 246 clinical samples were detected: 4.88% were positive for Cp, 2.44% for Ss, and 15.85% for To, while 0.81% for co-infections of Cp/To. This TaqMan probe-based pentaplex qPCR enables rapid detection and differential identification of five pathogens from clinical samples, eliminating the need for multiple single-plex qPCR. It can potentially assist clinicians in diagnosis and treatment planning by rapidly detecting pathogens in clinical samples and further quantitative analysis. It is of extremely significant importance for protecting human health.

## 1 Introduction

*Cryptosporidium parvum, Strongyloides stercoralis, Trypanosoma cruzi, Theileria ovis, Echinococcus granulosus* can cause diarrhea, fever, pneumonia, hepatitis, encephalitis, cardiovascular, and gastrointestinal disorders, as well as reproductive system diseases in humans and animals, posing a serious threat to human health, animal quality, and public health security. *Cryptosporidium parvum* is a pathogenic protozoan that causes diseases in humans. Its clinical symptoms range from mild to severe diarrhea, accompanied by complications such as developmental delays (Zhao et al., 2024; Sabir et al., 2021; Messa et al., 2021; Hanieh et al., 2021). It can also lead to gastrointestinal disorders (Bellinzona et al., 2024), and is the most common cause of bile duct lesions in AIDS patients (Alsharif et al., 2024; Mohamed et al., 2022). It can also cause diarrhea in patients with hematological malignancies undergoing chemotherapy (Bahadorizadeh et al., 2024). *Strongyloides stercoralis* can cause disease when the larvae invade human body through the skin or mucosa, and can also infect host when host ingests its eggs (Corda et al., 2025; Gardini et al., 2024). *Strongyloides stercoralis* infection lacks characteristic manifestations, and its clinical symptoms are related to the immune, response of host and the degree of infection. Immunodeficient patients with underlying diseases or long-term treatment with glucocorticoids who infected with *Strongyloides stercoralis* are more likely to develop severe disease (Samsudin et al., 2024; Uslu et al., 2024; Tang et al., 2024). Infection with *Trypanosoma cruzi* is a complex zoonotic disease transmitted by more than 130 triatomine species and sustained by mammalian hosts in more than 70 genera, and infection with humans can lead to Chagas disease, and an estimated 20% to 30% of infected individuals develop potentially lethal cardiac or gastrointestinal disease, with serious consequences for public health and national economies (Venturini et al., 2023; Crisóstomo-Vázquez et al., 2024; Lima-Neiva et al., 2021; Bern et al., 2019; Abras et al., 2022). *Theileria ovis* is a blood protozoan transmitted by ticks. It can cause symptoms such as jaundice, tachycardia, fatigue, fever, decreased milk production, as well as miscarriage or stillbirth (Irfan et al., 2023; Almahallawi et al., 2024; Caglar et al., 2024). *Echinococcus granulosus* can cause cystic echinococcosis, a zoonotic parasitic infection that poses a significant public health risk (Aliste-Fernández and José, 2020; Celik et al., 2025; Zarrabi Ahrabi et al., 2020). Therefore, it is of utmost significance to quickly and accurately detect pathogens such as *Cryptosporidium parvum, Strongyloides stercoralis, Trypanosoma cruzi, Theileria ovis*, and *Echinococcus granulosus*.

The most commonly used method for detecting parasitic pathogens is the microscopic examination. However, the effectiveness of direct microscopic examination may be affected by various factors such as the disease stage, clinical manifestations, the number of parasites, and other variables that significantly influence the diagnostic outcome. Additionally, the sensitivity of microscopic examination is relatively low, and the specificity of the test mainly depends on the subjective judgment of the microscope operator's experience and skills. This may lead to misjudgments of some less obvious morphological characteristics of parasites and improper treatment of the condition, thereby potentially causing environmental pollution and endangering the safety of the inspectors (Ndao, 2009). On the other hand, patients with chronic or latent parasitic pathogen infections pose greater diagnostic challenges because their parasite numbers are low or reduced, which may result in false negative results, delayed treatment, and poor patient prognosis.

In immunological methods, detecting pathogen-specific antibodies is the main diagnostic approach (Urrea-Quezada et al., 2022; De Souza et al., 2021; Carlier et al., 2021; Tao et al., 2022). However, this method is operationally complex or has issues regarding specificity and sensitivity. Moreover, for immunosuppressed patients, serological testing may yield false negative or false positive results due to the individual's immune status or the recurrence of Chagas disease (Veletzky et al., 2024; Moreira and Renan Cunha-Melo, 2020; Ringer et al., 2021; Clark et al., 2024). Serological testing often has some serious flaws, such as cross-reactions with other organisms closely related to the research subjects, and even after successful treatment, antibodies may persist for a long time, both of which can lead to false positive test results.

In nucleic acid amplification testing (NAAT), polymerase chain reaction (PCR) is the most commonly used molecular diagnostic technique and is currently regarded as the gold standard (Bailly et al., 2025; Blaizot et al., 2019; Benvenuti et al., 2021; Habibi et al., 2024). It significantly enhances the sensitivity and specificity of the test, even in samples with low or reduced parasite loads. However, PCR operations are cumbersome and time-consuming because the agarose gel electrophoresis and the screening process of the amplification products require a lot of time and effort. Moreover, the sensitivity of PCR is relatively low. Additionally, due to various factors such as opening test tubes, PCR is prone to contamination and is also susceptible to the influence of inhibitors that may exist in the samples.

The fluorescence quantitative real-time PCR (qPCR) based on TaqMan probes is a highly promising tool that can be used to detect and quantify the target genomic sequences of infectious pathogens in various samples (blood, tissue, body fluids, feces). Its fundamental principle lies in achieving this goal by continuously measuring the cumulative fluorescence signals during the amplification reaction process. Due to the fact that this technology has the advantages of simple operation, high speed, convenience, high sensitivity, good repeatability, and low contamination rate, it has been widely applied in fields such as medical testing, drug efficacy evaluation, gene expression research, transgenic research, gene detection, animal and plant testing, food testing, and pathogen detection. For instance, it has been previously reported that TaqMan probe-based single-plex qPCR can be used to detect *Cryptosporidium parvum* (Fontaine and Guillot, 2002), *Strongyloides stercoralis* (Verweij et al., 2009), *Trypanosoma cruzi* (Ramírez et al., 2015), *Echinococcus granulosus* (Maksimov et al., 2020), *Dictyocaulus filaria* (Gao and Xing, 2025), and *Clostridium piliforme* (Gao et al., 2012).

The multiplex qPCR technique involves detecting multiple target genes in a single test tube. To distinguish the fluorescence signals of these target genes, using only SYBR Green dye to label the amplification products is not sufficient to achieve this goal. Therefore, the TaqMan probe-based method is usually employed for multiplex qPCR. That is to say, for each target gene, a pair of primers and a probe will be designed, and by utilizing the fluorescent groups labeled on different probes and combining the detection capabilities of different channels of the instrument, it is feasible to conduct quantitative detection of multiple target genes of pathogens using TaqMan probe-based multiplex qPCR.

The mixed infection of *Cryptosporidium, Strongyloides stercoralis, Trypanosoma cruzi, Theileria ovis*, and *Echinococcus granulosus* is a serious problem. These pathogens are prone to mixed or secondary infections, making clinical differentiation extremely difficult. Therefore, developing a sensitive, specific and efficient detection method to simultaneously distinguish *Cryptosporidium parvum, Strongyloides stercoralis, Trypanosoma cruzi, Theileria ovis*, and *Echinococcus granulosus* is of vital importance for formulating targeted prevention and treatment strategies. To address these challenges, we have developed a TaqMan probe-based pentaplex qPCR method targeting the the *gp60* gene of *Cryptosporidium parvum*, the *COX1* gene of *Strongyloides stercoralis*, the *SAT* gene of *Trypanosoma cruzi*, the *SSU rRNA* gene of *Theileria ovis*, and the *ND1* gene of *Echinococcus granulosus*. The mechanism of this probably nice breakthrough is: In the same reaction system, five sets of specific primers and probes combinations were added. Each probe is labeled with different fluorescent dyes that do not interfere with each other. During pentaplex qPCR amplification, the Taq enzyme hydrolyzes the probe through its 5′ exonuclease activity, causing the reporter fluorescence to separate from the quenching group and thereby generating light. The instrument collects fluorescence signals at different wavelengths in five independent optical channels in qPCR to achieve simultaneous specific amplification and quantitative detection of five targets within a single tube. This TaqMan probe-based pentaplex qPCR method can accurately diagnose and distinguish the pathogens in clinical samples, which may provide an efficient and convenient strategy for disease prevention and control.

## 2 Materials and methods

### 2.1 Design of probes/primers and preparing of plasmid

The sequences of the *gp60* gene of *Cryptosporidium parvum* (GenBank: MW996761), the *COX1* gene of *Strongyloides stercoralis* (GenBank ID: AB526297), the *SAT* gene of *Trypanosoma cruzi* (GenBank ID: KT369012), the *SSU rRNA* gene of *Theileria ovis* (GenBank ID: MZ220429), and the *ND1* gene of *Echinococcus granulosus* (GenBank ID: KJ162560) were aligned with target sequences using the National Center for Biotechnology Information (NCBI) Basic Local Alignment Search Tool (BLAST) (https://blast.ncbi.nlm.nih.gov/Blast.cgi). Sequences showing high similarity with the target genes were downloaded and analyzed for specific and conserved regions using DNASTAR software (7.1 version) and Primer Express Software Version 3.0 (Thermo Fisher Scientific, Waltham, MA, USA). The conserved regions were selected for the synthesis of plasmid standards: pCpgp60, pSsCOX1, pTcSAT, pToSSUrRNA and pEgND1, and the copy number of them were calculated with the formula {plasmid copy number/μL= [6.02 × 10^23^ × plasmid concentration (ng/μL) × 10^−9^]/ [plasmid length × 660]} (Cohen et al., 2009). For the TaqMan probe-based pentaplex qPCR, specific primers and probes were designed. The 5′ ends of these probes were respectively labeled with fluorescent reporter groups such as FAM, VIC, ABY, JUN, and CY5, while the 3′ ends were respectively labeled with corresponding groove binding agents (MGB), non-fluorescent quenchers (NFQ) or QSY. Through experimental screening, primers and probes with strong specificity and high sensitivity were determined (Table 1).

**Table 1:** TaqMan probes and pentaplex qPCR primers^#^ used in this study.

Pathogen	Gene	Primer/probe	Sequence (5^′^end to 3^′^end)	Product size
*Cryptosporidium parvum*	*gp60*	GZQCpgp60-F	AGGCGCAACTACCGAAACC	62 bp
		GZQCpgp60-R	TTACAAATGAAGTGCCGCATTC	
		GZQCpgp60-P	(FAM)–TAGAAGCTACTCCAAAAGA–(MGB–NFQ)	
*Strongyloides stercoralis*	*COX1*	GZQSsCOX1-F	TTTGTTGATAATGGTTTGGGTACTAGTT	76 bp
		GZQSsCOX1-R	CACTAGAACCAGGATGACCTGAAGTA	
		GZQSsCOX1-P	(VIC)–AACAATTTATCCTCCTCTATC–(MGB–NFQ)	
*Trypanosoma cruzi*	*SAT*	GZQTcSAT-F	CGAGCGGCTGCTACATCA	120 bp
		GZQTcSAT-R	TTGTTTGGTGTCCAGTGTGTGA	
		GZQTcSAT-P	(ABY)–AGGCACTCTCTGTCACTATCTGTTTGCGTG–(QSY)	
*Theileria ovis*	*SSU rRNA*	GZQToSSUrRNA-F	GAGTATCAATTGGAGGGCAAGTCT	97 bp
		GZQToSSUrRNA-R	AACTACGAGCTTTTTAACTGCAACAA	
		GZQToSSUrRNA-P	(JUN)–CAGCAGCCGCGGTAATTCCAGC–(QSY)	
*Echinococcus granulosus*	*ND1*	GZQEgND1-F	CGGTTCGATGTGCTTTTGG	68 bp
		GZQEgND1-R	AGCACAAAAAATCACCACACACATA	
		GZQEgND1-P	(CY5)–TCTGTTAGGTTTGAGGCTT–(MGB–NFQ)	

^#^All TaqMan probes and pentaplex qPCR primers are protected by patent. For any commercial use please contact the corresponding author.

### 2.2 Pathogens and clinical samples used

*Toxoplasma gondii, Giardia lamblia, Balantidium coli, Aspiculuris tetraptera, Syphacia obvelata*, and *Syphacia muris* were maintained in our laboratory. Clinical samples were preserved at −80°C in this laboratory. These blood and tissue samples were mainly collected in Beijing, Jilin Province and Shandong Province of China. The DNA stored at −20°C was used as a template for the PCR. All positive samples were identified in our laboratory using the PCR method, and confirmed using DNA sequencing (Takara Biomedical Technology Co., Ltd., Beijing, China).

### 2.3 Extraction of DNA

According to the manufacturer′s instructions, total DNA was extracted from clinical samples using the DNeasy Blood & Tissue Kit (Qiagen, Germany). Pathogenic DNA was extracted using the QIAamp DNA Mini Kit (Qiagen, Germany). The purity, yield and extraction efficiency (*EE*) of DNA were monitored by using the Xeno Internal Positive Control (IPC) DNA spiking.

### 2.4 Optimization of the TaqMan probe-based pentaplex qPCR

The primer and probe concentration, and annealing temperatures of the TaqMan probe-based pentaplex qPCR were optimized to set up the optimal reaction system and conditions for the simultaneous detection of *Cryptosporidium parvum, Strongyloides stercoralis, Trypanosoma cruzi, Theileria ovis*, and *Echinococcus granulosus*. Briefly, the TaqMan probe-based pentaplex qPCR reactions were conducted in a 20-μL volume, which included 10 μL of TaqPath™ ProAmp™ Multiplex Master Mix (Thermo Fisher Scientific, USA), 3 μL of Primers/Probes Mix (0.22 μM of forward primer GZQCpgp60-F, 0.22 μM of reverse primer GZQCpgp60-R, and 0.06 μM of TaqMan probe GZQCpgp60-P for *Cryptosporidium parvum*, 0.22 μM of forward primer GZQpSsCOX1-F, 0.22 μM of reverse primer GZQSsCOX1-R, and 0.06 μM of TaqMan probe GZQ SsCOX1-P for *Strongyloides stercoralis*, 0.22 μM of forward primer GZQTcSAT-F, 0.22 μM of reverse primer GZQTcSAT-R, and 0.06 μM of TaqMan probe GZQTcSAT-P for *Trypanosoma cruzi*, 0.22 μM of forward primer GZQToSSUrRNA-F, 0.22 μM of reverse primer GZQToSSUrRNA-R, and 0.06 μM of TaqMan probe GZQToSSUrRNA-P for *Theileria ovis*, and 0.44 μM of forward primer GZQEgND1-F, 0.44 μM of reverse primer GZQEgND1-R, and 0.12 μM of TaqMan probe GZQEgND1-P for *Echinococcus granulosus* in final concentration), 5 μL of template DNA, and 2 μL of nuclease-free water. The amplification reaction was performed on the QuantStudio™ 6 Real-time PCR System. The amplification steps were as follows: 50°C for 2 min (enzyme activation); 95°C for 10 min (initial denaturation); then 40 cycles were carried out, with each cycle consisting of 15 s at 95°C (denaturation) and 1 min at 60°C (annealing). At the end of each cycle, the fluorescence signal intensities of FAM, VIC, ABY, JUN and CY5 were recorded. Through automatic threshold setting, the quantification cycle (Cq) values were generated using the QuantStudio Design & Analysis Software.

### 2.5 Efficiency, lower limit of quantification, and limit of detection of the TaqMan probe-based pentaplex qPCR

In order to evaluate the linearity, dynamic range, amplification efficiency (*E*), lower limit of quantification (LLOQ), and limit of detection (LOD) of the TaqMan probe-based pentaplex qPCR (Bustin et al., 2009; Forootan et al., 2017; Ruijter et al., 2021; Bustin et al., 2025), standard DNA samples of *Cryptosporidium parvum, Strongyloides stercoralis, Trypanosoma cruzi, Theileria ovis*, and *Echinococcus granulosus* were serially diluted and used as templates for testing. Based on the Cq values and the logarithms of standard copy numbers, a standard curve was ultimately generated (Rutledge and Côté, 2003). By plotting a graph of Cq (on the vertical axis) vs. the logarithm of the target concentration (on the horizontal axis), the slope of the linear regression can be determined. This amplification efficiency (*E*) is determined by the slope, and its calculation formula is: %*E*=100 × (−1+10^(−1/*slope*)^).

### 2.6 Specificity of the TaqMan probe-based pentaplex qPCR

DNA was extracted from *Toxoplasma gondii, Giardia lamblia, Balantidium coli, Aspiculuris tetraptera, Syphacia obvelata*, and *Syphacia muris*, as non-target genes. Recombinant plasmid standards of *Cryptosporidium parvum, Strongyloides stercoralis, Trypanosoma cruzi, Theileria ovis*, and *Echinococcus granulosus* were used as positive controls, while the nuclease-free water was used as the no-template control (NTC), and the XenoInternal Positive Control – VIC Assay was used as an internal control (IPC). The samples were tested using the developed TaqMan probe-based pentaplex qPCR assay.

### 2.7 Repeatability and reproducibility of TaqMan probe-based pentaplex qPCR

The recombinant plasmid standards pCpgp60, pSsCOX1, pTcSAT, pToSSUrRNA, and pEgND1 were diluted from each reaction concentration of 5 × 10^7^ copies to 5 × 10^3^ copies using a gradient of TE buffer (Thermo Fisher Scientific, USA). Then, these diluted solutions were mixed in equal volumes and subjected to an optimized TaqMan probe-based pentaplex qPCR, ultimately diluting the concentration range from each reaction of 1 × 10^7^ copies to 1 × 10^3^ copies. On the same day, the same dilution operation was performed three times for each type of DNA to determine the intra group repeatability, and on three different dates within a week, each type of DNA was tested independently three times to determine the inter group repeatability. The standard deviation (SD) value and coefficient of variation (CV) of the Cq values were calculated using the formula CV= (standard deviation / average value) × 100%.

### 2.8 Anti-interference test of TaqMan probe-based pentaplex qPCR

The plasmid concentrations of 1 × 10^8^ copies and 1 × 10^2^ copies were selected respectively, and the standard plasmid concentrations of *Cryptosporidium parvum, Strongyloides stercoralis, Trypanosoma cruzi, Theileria ovis*, and *Echinococcus granulosus* were randomly combined together. Three parallel samples were prepared for each experiment to observe the changes in the Cq values at low concentrations and to assess whether high concentrations would affect amplification at low concentrations using the developed TaqMan probe-based pentaplex qPCR assay.

### 2.9 Simulation of mixed infection by combining different concentration of standard samples using TaqMan probe-based pentaplex qPCR

At different concentrations, DNA standards of *Cryptosporidium parvum, Strongyloides stercoralis, Trypanosoma cruzi, Theileria ovis*, and *Echinococcus granulosus* were randomly selected and mixed together as templates. Then, they were detected using the developed TaqMan probe-based pentaplex qPCR assay.

### 2.10 Validation of TaqMan probe-based pentaplex qPCR

For 16 clinical samples with diarrhea symptoms, both TaqMan probe-based pentaplex qPCR and PCR were employed for testing simultaneously. The PCR primers were synthesized by Takara Biomedical Technology Co., Ltd., Beijing, China (Table 2). The total volume of the PCR reaction was 50 μL, consisting 5 μL of 10 × Buffer (Mg^2+^ Plus), 4 μL of dNTP Mixture, 0.5 μL of forward primer (10 μM), 0.5 μL of reverse primer (10 μM), 0.25 μL of EX Taq (5 U/μL) (Takara Biomedical Technology Co., Ltd., Beijing, China), 2 μL of template DNA, and 37.75 μL of nuclease-free water. The amplification operation was performed using the Verity 96 Thermal Cycler Instrument (Applied Biosystems, USA). The amplification procedure was as follows: 94°C for 5 min, 1 cycle; 95°C for 30 s, 60°C for 30 s, 72°C for 1 min, a total of 35 cycles; 72°C for 10 min, 1 cycle. The PCR amplification products were detected using electrophoresis on 3% agarose gel and visualized under the UV light after nucleic acid staining. The detection results of the TaqMan probe-based pentaplex qPCR were compared with those of the PCR.

**Table 2:** PCR primers used in this study.

Pathogen	Gene	Primer	Sequence (5^′^end to 3^′^end)	Product size
*Cryptosporidium parvum*	*SSU rRNA*	GZQCpSSUrRNA-CF	GACAGTTGGGGGCATTTGTATT	124 bp
		GZQCpSSUrRNA-CR	CGATCCCCTAACTTTCGTTCTTGA	
*Strongyloides stercoralis*	*COX1*	GZQSsCOX1-CF	TGGTTTGGGTACTAGTTGAACA	75 bp
		GZQSsCOX1-CR	GCCAAATCAACACTAGAACCAGG	
*Trypanosoma cruzi*	*SAT*	GZQTcSAT-CF	AGTCGGCTGATCGTTTTCG	160 bp
		GZQTcSAT-CR	AATTCCTCCAAGCAGCGGAT	
*Theileria ovis*	*SSU rRNA*	GZQToSSUrRNA-CF	ATTGGAGGGCAAGTCTGGTG	138 bp
		GZQToSSUrRNA-CR	CCACAATGCAAAGACTCGT	
*Echinococcus granulosus*	*RBP*	GZQEgND1-CF	ATTTGCGATTCCGAGAGCTG	115 bp
		GZQEgND1-CR	CCACGCTGAATGGCACTTTC	

### 2.11 Clinical performance of TaqMan probe-based pentaplex qPCR

Using the TaqMan probe-based pentaplex qPCR developed in this study, the detection rates of *Cryptosporidium parvum, Strongyloides stercoralis, Trypanosoma cruzi, Theileria ovis*, and *Echinococcus granulosus* in 16 diarrhea samples and 230 samples without any symptoms were studied. Nucleic acids were extracted using the DNA extraction kit. The constructed plasmids were used as the positive controls, the nuclease-free water was used as the no-template control (NTC), and the XenoInternal Positive Control – VIC Assay as an internal control (IPC). After obtaining the detection results of all clinical samples, the detection rates were analyzed.

### 2.12 Statistics analyses

As mentioned above, the statistical analysis was conducted using GraphPad Prism 8.0 software. All experiments were repeated at least three times and the results were similar. The data were presented in the form of mean ± standard deviation, unless otherwise stated. The correlation coefficient values and amplification efficiency of the standard curve were calculated, and the TaqMan probe-based pentaplex qPCR amplification curves were visualized using QuantStudio Design & Analysis software (version: 1.4.1).

## 3 Results

### 3.1 Efficiency of the TaqMan probe-based pentaplex qPCR

**Figure 1:**
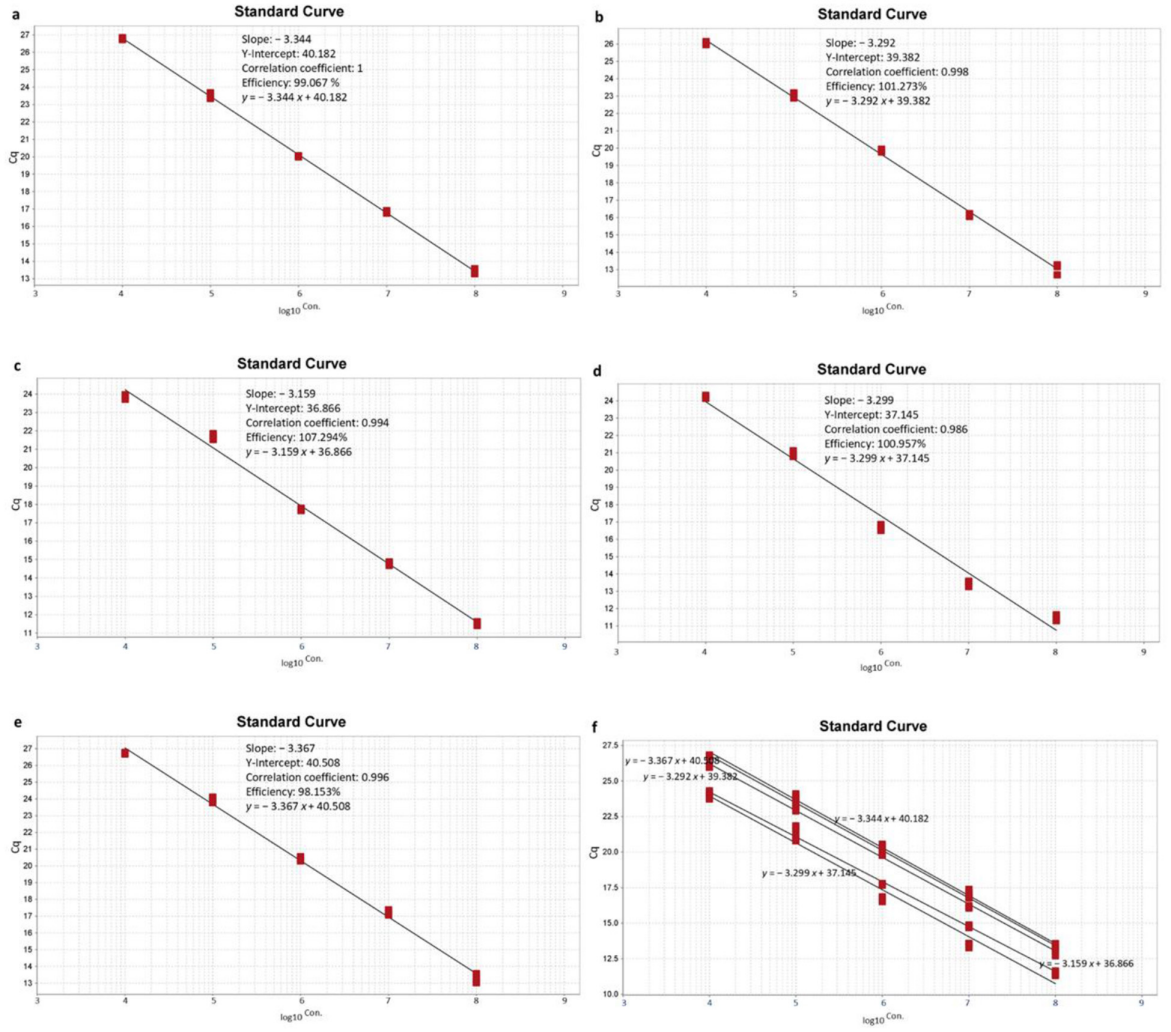
Standard curves of the TaqMan probe-based pentaplex qPCR for simultaneously detecting *Cryptosporidium parvum, Strongyloides stercoralis, Trypanosoma cruzi, Theileria ovis*, and *Echinococcus granulosus*. **(a)** Standard curve of *Cryptosporidium parvum* using the TaqMan probe-based pentaplex qPCR. The standard curve of *Cryptosporidium parvum* for DNA standards at concentrations ranging from 1 × 10^8^ to 1 × 10^4^ copies per reaction. **(b)** Standard curve of *Cryptosporidium parvum* using the TaqMan probe-based pentaplex qPCR. The standard curve of *Strongyloides stercoralis* for DNA standards at concentrations ranging from 1 × 10^8^ to 1 × 10^4^ copies per reaction. **(c)** Standard curve of *Trypanosoma cruzi* using the TaqMan probe-based pentaplex qPCR. The standard curve of *Trypanosoma cruzi* for DNA standards at concentrations ranging from 1 × 10^8^ to 1 × 10^4^ copies per reaction. **(d)** Standard curve of *Theileria ovis* using the TaqMan probe-based pentaplex qPCR. The standard curve of *Theileria ovis* for DNA standards at concentrations ranging from 1 × 10^8^ to 1 × 10^4^ copies per reaction. **(e)** Standard curve of *Echinococcus granulosus* using the TaqMan probe-based pentaplex qPCR. The standard curve of *Echinococcus granulosus* for DNA standards at concentrations ranging from 1 × 10^8^ to 1 × 10^4^ copies per reaction. **(f)** Standard curves of *Cryptosporidium parvum, Strongyloides stercoralis, Trypanosoma cruzi, Theileria ovis*, and *Echinococcus granulosus* using the TaqMan probe-based pentaplex qPCR. Standard curves of *Cryptosporidium parvum, Strongyloides stercoralis, Trypanosoma cruzi, Theileria ovis*, and *Echinococcus granulosus* for DNA standards at concentrations ranging from 1 × 10^8^ to 1 × 10^4^ copies per reaction.

The standard curves were plotted, where the horizontal axis represents the logarithm of the amount of the initial template, and the vertical axis represents the Cq value. Standard curves were established for *Cryptosporidium parvum, Strongyloides stercoralis, Trypanosoma cruzi, Theileria ovis*, and *Echinococcus granulosus*. The correlation coefficients (*R*^2^) were 1, 0.998, 0.994, 0.986, and 0.996 respectively. The slopes of the equations were −3.344, −3.292, −3.159, −3.299, and −3.367 respectively. The intercepts were 40.182, 39.382, 36.866, 37.145, and 40.508 respectively. The amplification efficiencies (*E*) were 99.067%, 101.273%, 107.294%, 100.957%, and 98.153% respectively. As shown in Figure 1, the standard formulas for *Cryptosporidium parvum, Strongyloides stercoralis, Trypanosoma cruzi, Theileria ovis*, and *Echinococcus granulosus* were as follows: *y* = −3.344*x* + 40.182, *y* = −3.292*x* + 39.382, *y* = −3.159*x* + 36.866, *y* = −3.299*x* + 37.145, and *y* = −3.367*x* + 40.508 respectively. Figure 1 also showed that the fluorescence signals were detected in three parallel samples for each concentration gradient of these five target pathogens. The results demonstrated the reliability of the TaqMan probe-based pentaplex qPCR for quantitative analysis of nucleic acids in amplified samples. Since the correlation coefficient value is close to 1 and the amplification efficiency is within the optimal range of 90% to 110%, these results indicate an excellent linear relationship between the template DNA concentrations and the corresponding Cq values.

### 3.2 Specificity and lower limit of quantification of the TaqMan probe-based pentaplex qPCR

**Figure 2:**
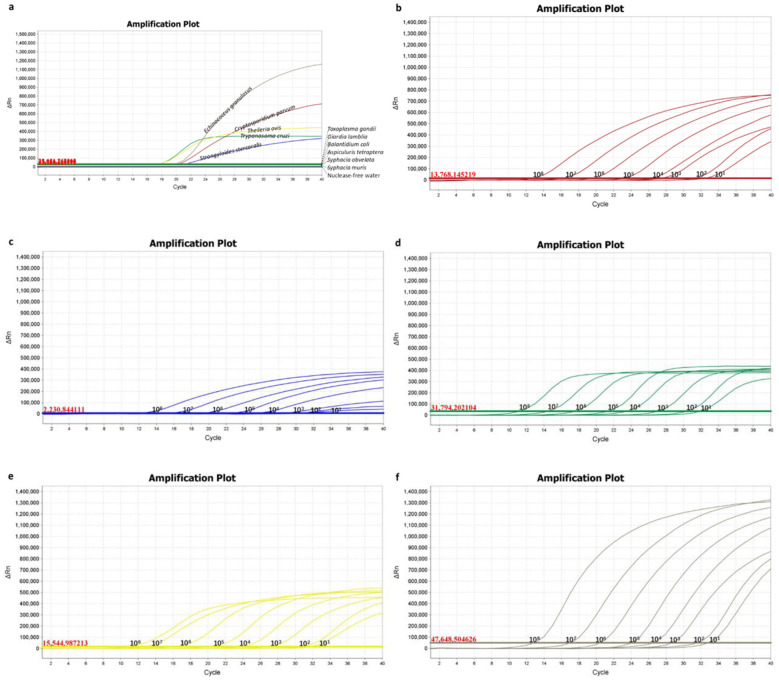
Specificity and lower limit of quantification (LLOQ) of the TaqMan probe-based pentaplex qPCR for simultaneously detecting *Cryptosporidium parvum, Strongyloides stercoralis, Trypanosoma cruzi, Theileria ovis*, and *Echinococcus granulosus*. **(a)** Amplification curves represented samples positive for *Cryptosporidium parvum, Strongyloides stercoralis, Trypanosoma cruzi, Theileria ovis*, and *Echinococcus granulosu*. Negative samples included *Toxoplasma gondii, Giardia lamblia, Balantidium coli, Aspiculuris tetraptera, Syphacia obvelata, Syphacia muris*. Nuclease-free water was used as no template control. **(b)** The amplification curves of *Cryptosporidium parvum* for DNA standards at concentrations ranging from 1 × 10^8^ to 1 × 10^1^ copies per reaction. **(c)** The amplification curves of *Strongyloides stercoralis* for DNA standards at concentrations ranging from 1 × 10^8^ to 1 × 10^1^ copies per reaction. **(d)** The amplification curves of *Trypanosoma cruzi* for DNA standards at concentrations ranging from 1 × 10^8^ to 1 × 10^1^ copies per reaction. **(e)** The amplification curves of *Theileria ovis* for DNA standards at concentrations ranging from 1 × 10^8^ to 1 × 10^1^ copies per reaction. **(f)** The amplification curves of *Echinococcus granulosus* for DNA standards at concentrations ranging from 1 × 10^8^ to 1 × 10^1^ copies per reaction.

As shown in Figure 2, *Cryptosporidium parvum, Strongyloides stercoralis, Trypanosoma cruzi, Theileria ovis*, and *Echinococcus granulosus* all produced typical amplification curves, while *Toxoplasma gondii, Giardia lamblia, Balantidium coli, Aspiculuris tetraptera, Syphacia obvelata*, and *Syphacia muris* did not show amplification curves or Cq values. The results indicated that the developed TaqMan probe-based pentaplex qPCR had strong specificity.

The developed TaqMan probe-based pentaplex qPCR was used to test a 10-fold dilution gradient of the DNA standard. Figure 2 also showed that the lower limit of quantification (LLOQ) of this method was 10 copies per reaction, and it could be used to simultaneously detect *Cryptosporidium parvum, Strongyloides stercoralis, Trypanosoma cruzi, Theileria ovis*, and *Echinococcus granulosus*. The results indicated that the TaqMan probe-based pentaplex qPCR had high sensitivity.

### 3.3 Limit of detection of the TaqMan probe-based pentaplex qPCR

**Figure 3:**
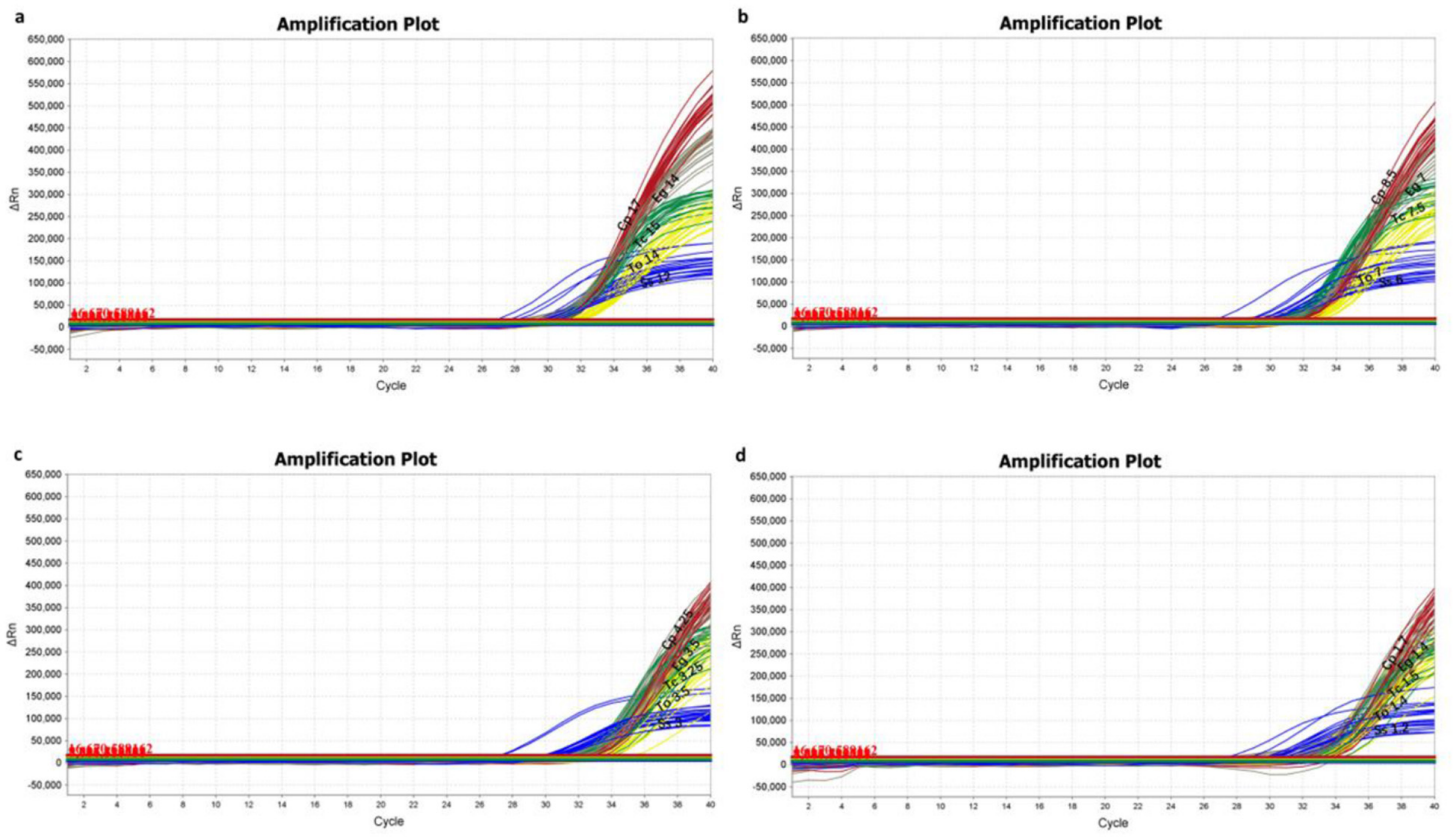
Limit of detection (LOD) of the TaqMan probe-based pentaplex qPCR for simultaneously detecting *Cryptosporidium parvum, Strongyloides stercoralis, Trypanosoma cruzi, Theileria ovis*, and *Echinococcus granulosus*. **(a)** Amplification curves of *Cryptosporidium parvum, Strongyloides stercoralis, Trypanosoma cruzi, Theileria ovis*, and *Echinococcus granulosus* using serial dilutions of DNA standards 17, 12, 15, 14, and 14 copies per reaction. **(b)** Amplification curves of *Cryptosporidium parvum, Strongyloides stercoralis, Trypanosoma cruzi, Theileria ovis*, and *Echinococcus granulosus* using serial dilutions of DNA standards 8.5, 6, 7.5, 7, and 7 copies per reaction. **(c)** Amplification curves of *Cryptosporidium parvum, Strongyloides stercoralis, Trypanosoma cruzi, Theileria ovis*, and *Echinococcus granulosus* using serial dilutions of DNA standards 4.25, 3, 3.25, 3.5, and 3.5 copies per reaction. **(d)** Amplification curves of *Cryptosporidium parvum, Strongyloides stercoralis, Trypanosoma cruzi, Theileria ovis*, and *Echinococcus granulosus* using serial dilutions of DNA standards 1.7, 1.2, 1.5, 1.4, and 1.4 copies per reaction.

The two-fold dilution of the DNA standard was tested using the developed TaqMan probe-based pentaplex qPCR (Table 3). The limit of detection (LOD) of this experiment was defined as the lowest concentration of the target analyte that could be detected with a 95% detection rate (Figure 3). Subsequent experiments demonstrated that the detection rates for samples of *Cryptosporidium parvum* (1.7 copies per reaction), *Strongyloides stercoralis* (1.2 copies per reaction), *Trypanosoma cruzi* (1.5 copies per reaction), *Theileria ovis* (1.4 copies per reaction), and *Echinococcus granulosus* (1.4 copies per reaction) were 100%, 100%, 100%, 95%, and 100% respectively for the results of 20 replicates. Therefore, the reliable LODs of the TaqMan probe-based pentaplex qPCR were 1.7, 1.2. 1.5. 1.4, and 1.4 copies per reaction for the simultaneous detection of *Cryptosporidium parvum, Strongyloides stercoralis, Trypanosoma cruzi, Theileria ovis*, and *Echinococcus granulosus*.

**Table 3:** Limit of detection (LOD) of the TaqMan probe-based pentaplex qPCR.

Pathogen	Con. (copies per reaction)	Repeat times	Positive number	Positive rate	95% positive rate
*Cryptosporidium parvum*	17	20	20	100%	>95%
	8.5	20	20	100%	>95%
	4.25	20	20	100%	>95%
	1.7	20	20	100%	>95%
*Strongyloides stercoralis*	12	20	20	100%	>95%
	6	20	20	100%	>95%
	3	20	20	100%	>95%
	1.2	20	20	100%	>95%
*Trypanosoma cruzi*	15	20	20	100%	>95%
	7.5	20	20	100%	>95%
	3.25	20	20	100%	>95%
	1.5	20	20	100%	>95%
*Theileria ovis*	14	20	20	100%	>95%
	7	20	20	100%	>95%
	3.5	20	20	100%	>95%
	1.4	20	19	95%	=95%
*Echinococcus granulosus*	14	20	20	100%	>95%
	7	20	20	100%	>95%
	3.5	20	20	100%	>95%
	1.4	20	20	100%	>95%

### 3.4 Repeatability and reproducibility of the TaqMan probe-based pentaplex qPCR

As shown in Table 4, the intra group coefficient of variation of the developed TaqMan probe-based pentaplex qPCR for simultaneously detecting *Cryptosporidium parvum* ranged from 0.11% to 0.60%, *Strongyloides stercoralis* from 0.09% to 1.18%, *Trypanosoma* cruzi from 0.77% to 1.34%, *Theileria ovis* from 0.22% to 0.77% and *Echinococcus granulosus* from 0.15% to 0.72%.

**Table 4:** Repeatability and reproducibility of the TaqMan probe-based pentaplex qPCR.

Pathogen	Con. (copies per reaction)	Intra-assay		Inter-assay	
		Cq value (mean ±SD)	CV	Cq value (mean ±SD)	CV
*Cryptosporidium parvum*	1 × 10^7^	16.07 ± 0.098	0.60%	16.26 ± 0.084	0.51%
	1 × 10^6^	19.22 ± 0.066	0.34%	19.55 ± 0.068	0.34%
	1 × 10^5^	22.72 ± 0.072	0.31%	24.36 ± 0.099	0.40%
	1 × 10^4^	25.95 ± 0.029	0.11%	26.95 ± 0.067	0.24%
	1 × 10^3^	29.85 ± 0.095	0.31%	30.02 ± 0.23	0.76%
*Strongyloides stercoralis*	1 × 10^7^	17.36 ± 0.14	0.85%	16.39 ± 0.44	2.73%
	1 × 10^6^	20.76 ± 0.24	1.18%	20.28 ± 0.34	1.67%
	1 × 10^5^	23.94 ± 0.095	0.39%	23.04 ± 0.61	2.65%
	1 × 10^4^	27.59 ± 0.027	0.09%	25.78 ± 0.59	2.28%
	1 × 10^3^	31.00 ± 0.15	0.48%	30.13 ± 0.31	1.04%
*Trypanosoma cruzi*	1 × 10^7^	15.80 ± 0.17	1.07%	15.72 ± 0.09	0.57%
	1 × 10^6^	19.38 ± 0.15	0.77%	18.44 ± 0.11	0.59%
	1 × 10^5^	22.29 ± 0.30	1.34%	22.32 ± 0.12	0.53%
	1 × 10^4^	25.52 ± 0.23	0.90%	25.41 ± 0.084	0.33%
	1 × 10^3^	28.75 ± 0.24	0.83%	28.13 ± 0.20	0.71%
*Theileria ovis*	1 × 10^7^	14.12 ± 0.10	0.70%	13.76 ± 0.11	0.79%
	1 × 10^6^	17.98 ± 0.14	0.77%	17.13 ± 0.099	0.57%
	1 × 10^5^	21.50 ± 0.068	0.31%	20.53 ± 0.12	0.58%
	1 × 10^4^	24.91 ± 0.055	0.22%	24.69 ± 0.13	0.52%
	1 × 10^3^	27.84 ± 0.11	0.39%	28.34 ± 0.085	0.29%
*Echinococcus granulosus*	1 × 10^7^	16.61 ± 0.12	0.72%	16.82 ± 0.12	0.71%
	1 × 10^6^	19.92 ± 0.03	0.15%	20.16 ± 0.036	0.17%
	1 × 10^5^	24.69 ± 0.14	0.56%	24.57 ± 0.038	0.15%
	1 × 10^4^	27.23 ± 0.10	0.36%	27.36 ± 0.097	0.35%
	1 × 10^3^	30.33 ± 0.20	0.65%	30.76 ± 0.19	0.61%

Table 4 displayed that the coefficient of variation of the inter group of the developed TaqMan probe-based pentaplex qPCR for simultaneously detecting *Cryptosporidium parvum* ranged from 0.24% to 0.76%, *Strongyloides stercoralis* from 1.04% to 2.73%, *Trypanosoma cruzi* from 0.33% to 0.71%, *Theileria ovis* from 0.29% to 0.79%, *Echinococcus granulosus* from 0.15% to 0.71%. The results illuminated that both intra group and inter group coefficient of variations were less than 3%, suggesting excellent repeatability (intra group) and reproducibility (inter group) of the TaqMan probe-based pentaplex qPCR.

### 3.5 Anti-interference test of the TaqMan probe-based pentaplex qPCR

The TaqMan probe-based pentaplex qPCR amplification experiments were conducted for recombinant plasmids of *Cryptosporidium parvum, Strongyloides stercoralis, Trypanosoma cruzi, Theileria ovis*, and *Echinococcus granulosus*. The experimental conditions included high concentrations (1 × 10^8^ copies per reaction) and low concentrations (1 × 10^2^ copies per reaction). The presence of any potential interference was evaluated by observing the threshold changes in the amplification curves. As shown in Table 5, during the amplification process of any one of the high-concentration pathogens, no interference with the other four low-concentration pathogens was detected, and no interference with the low-concentration pathogens was found among any of the four high-concentration pathogens either.

**Table 5:** Anti-interference of the TaqMan probe-based pentaplex qPCR.

Item	*Cryptosporidium parvum*	*Strongyloides stercoralis*	*Trypanosoma cruzi*	*Theileria ovis*	*Echinococcus granulosus*
1	1 × 10^8^ copies per reaction	1 × 10^2^ copies per reaction	1 × 10^2^ copies per reaction	1 × 10^2^ copies per reaction	1 × 10^2^ copies per reaction
Cq value	12.56	33.01	31.09	31.62	32.93
2	1 × 10^2^ copies per reaction	1 × 10^8^ copies per reaction	1 × 10^2^ copies per reaction	1 × 10^2^ copies per reaction	1 × 10^2^ copies per reaction
Cq value	32.24	14.61	31.78	31.53	32.91
3	1 × 10^2^ copies per reaction	1 × 10^2^ copies per reaction	1 × 10^8^ copies per reaction	1 × 10^2^ copies per reaction	1 × 10^2^ copies per reaction
Cq value	32.41	33.08	12.93	31.98	32.80
4	1 × 10^2^ copies per reaction	1 × 10^2^ copies per reaction	1 × 10^2^ copies per reaction	1 × 10^8^ copies per reaction	1 × 10^2^ copies per reaction
Cq value	32.35	33.42	31.57	11.07	32.82
5	1 × 10^2^ copies per reaction	1 × 10^2^ copies per reaction	1 × 10^2^ copies per reaction	1 × 10^2^ copies per reaction	1 × 10^8^ copies per reaction
Cq value	32.59	33.16	31.19	31.76	12.74
6	1 × 10^2^ copies per reaction	1 × 10^8^ copies per reaction	1 × 10^8^ copies per reaction	1 × 10^8^ copies per reaction	1 × 10^8^ copies per reaction
Cq value	32.30	14.88	12.67	11.05	12.76
7	1 × 10^8^ copies per reaction	1 × 10^2^ copies per reaction	1 × 10^8^ copies per reaction	1 × 10^8^ copies per reaction	1 × 10^8^ copies per reaction
Cq value	12.62	33.16	12.56	10.92	12.06
8	1 × 10^8^ copies per reaction	1 × 10^8^ copies per reaction	1 × 10^2^ copies per reaction	1 × 10^8^ copies per reaction	1 × 10^8^ copies per reaction
Cq value	12.72	14.84	31.66	10.88	13.00
9	1 × 10^8^ copies per reaction	1 × 10^8^ copies per reaction	1 × 10^8^ copies per reaction	1 × 10^2^ copies per reaction	1 × 10^8^ copies per reaction
Cq value	12.87	14.69	12.97	31.31	13.03
10	1 × 10^8^ copies per reaction	1 × 10^8^ copies per reaction	1 × 10^8^ copies per reaction	1 × 10^8^ copies per reaction	1 × 10^2^ copies per reaction
Cq value	12.50	14.79	12.85	10.91	32.47

### 3.6 Assessment of mixed infection in the TaqMan probe-based pentaplex qPCR

**Figure 4:**
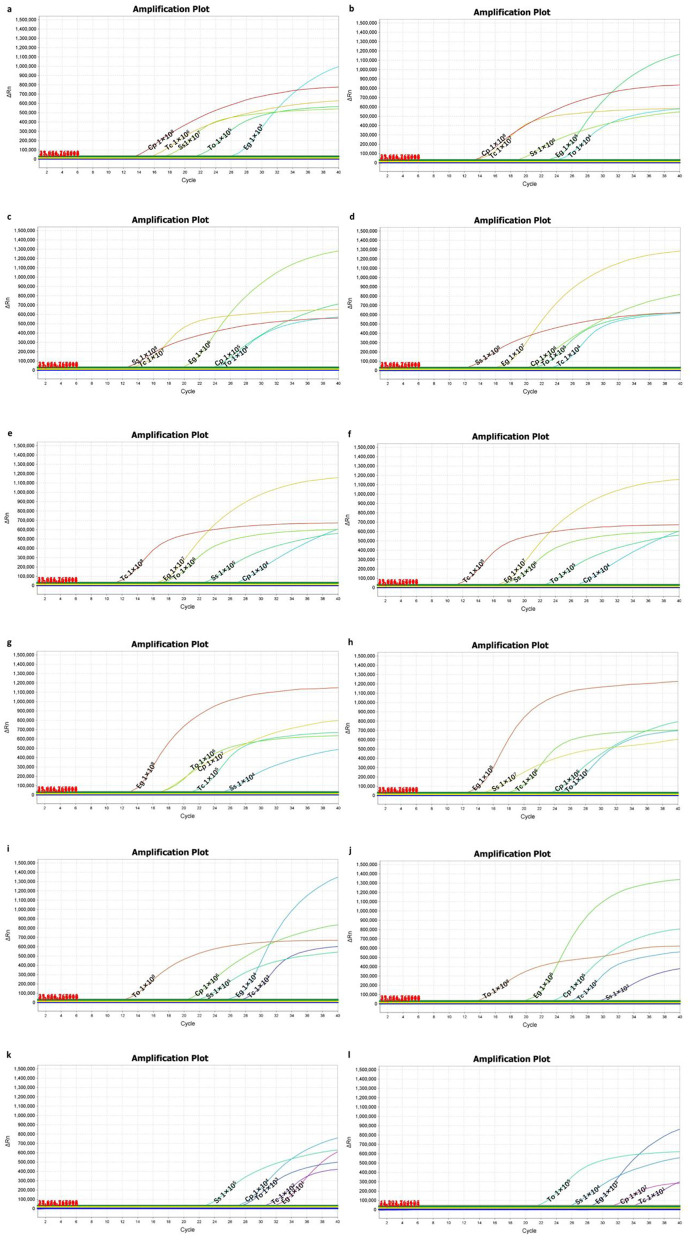
Mixed infection simulation experiments with *Cryptosporidium parvum* (Cp), *Strongyloides stercoralis* (Ss), *Trypanosoma cruzi* (Tc), *Theileria ovis* (To), and *Echinococcus granulosus* (Eg) using the TaqMan probe-based pentaplex qPCR. **(a)** Mixed infection amplification curves of *Cryptosporidium parvum, Strongyloides stercoralis, Trypanosoma cruzi, Theileria ovis*, and *Echinococcus granulosus* using serial dilutions of DNA standards 1 × 10^8^, 1 × 10^7^, 1 × 10^6^, 1 × 10^5^, and 1 × 10^4^ copies per reaction. **(b)** Mixed infection amplification curves of *Cryptosporidium parvum, Strongyloides stercoralis, Trypanosoma cruzi, Theileria ovis*, and *Echinococcus granulosus* using serial dilutions of DNA standards 1 × 10^8^, 1 × 10^6^, 1 × 10^7^, 1 × 10^4^, and 1 × 10^5^ copies per reaction. **(c)** Mixed infection amplification curves of *Cryptosporidium parvum, Strongyloides stercoralis*, Trypanosoma cruzi, *Theileria ovis*, and *Echinococcus granulosus* using serial dilutions of DNA standards 1 × 10^5^, 1 × 10^8^, 1 × 10^7^, 1 × 10^4^, and 1 × 10^6^ copies per reaction. **(d)** Mixed infection amplification curves of *Cryptosporidium parvum, Strongyloides stercoralis, Trypanosoma cruzi, Theileria ovis*, and *Echinococcus granulosus* using serial dilutions of DNA standards 1 × 10^6^, 1 × 10^8^, 1 × 10^4^, 1 × 10^5^, and 1 × 10^7^ copies per reaction. **(e)** Mixed infection amplification curves of *Cryptosporidium parvum, Strongyloides stercoralis, Trypanosoma cruzi, Theileria ovis*, and *Echinococcus granulosus* using serial dilutions of DNA standards 1 × 10^4^, 1 × 10^5^, 1 × 10^8^, 1 × 10^6^, and 1 × 10^7^ copies per reaction. **(f)** Mixed infection amplification curves of *Cryptosporidium parvum, Strongyloides stercoralis, Trypanosoma cruzi, Theileria ovis*, and *Echinococcus granulosus* using serial dilutions of DNA standards 1 × 10^4^, 1 × 10^6^, 1 × 10^8^, 1 × 10^5^, and 1 × 10^7^ copies per reaction. **(g)** Mixed infection amplification curves of *Cryptosporidium parvum, Strongyloides stercoralis, Trypanosoma cruzi, Theileria ovis*, and *Echinococcus granulosus* using serial dilutions of DNA standards 1 × 10^7^, 1 × 10^4^, 1 × 10^5^, 1 × 10^6^, and 1 × 10^8^ copies per reaction. **(h)** Mixed infection amplification curves of *Cryptosporidium parvum, Strongyloides stercoralis, Trypanosoma cruzi, Theileria ovis*, and *Echinococcus granulosus* using serial dilutions of DNA standards 1 × 10^5^, 1 × 10^7^, 1 × 10^6^, 1 × 10^4^, and 1 × 10^8^ copies per reaction. **(i)** Mixed infection amplification curves of *Cryptosporidium parvum, Strongyloides stercoralis, Trypanosoma cruzi, Theileria ovis*, and *Echinococcus granulosus* using serial dilutions of DNA standards 1 × 10^6^, 1 × 10^5^, 1 × 10^3^, 1 × 10^8^, and 1 × 10^4^ copies per reaction. **(j)** Mixed infection amplification curves of *Cryptosporidium parvum, Strongyloides stercoralis, Trypanosoma cruzi, Theileria ovis*, and *Echinococcus granulosus* using serial dilutions of DNA standards 1 × 10^5^, 1 × 10^3^, 1 × 10^4^, 1 × 10^8^, and 1 × 10^6^ copies per reaction. **(k)** Mixed infection amplification curves of *Cryptosporidium parvum, Strongyloides stercoralis, Trypanosoma cruzi, Theileria ovis*, and *Echinococcus granulosus* using serial dilutions of DNA standards 1 × 10^4^, 1 × 10^5^, 1 × 10^2^, 1 × 10^3^, and 1 × 10^1^ copies per reaction. **(l)** Mixed infection amplification curves of *Cryptosporidium parvum, Strongyloides stercoralis, Trypanosoma cruzi, Theileria ovis*, and *Echinococcus granulosus* using serial dilutions of DNA standards 1 × 10^2^, 1 × 10^4^, 1 × 10^1^, 1 × 10^5^, and 1 × 10^3^ copies per reaction.

As shown in Figure 4, the standard plasmids were used to simulate mixed infections commonly presented in clinical samples in mixed infection analysis. The results indicated that the TaqMan probe-based pentaplex qPCR was capable of detecting *Cryptosporidium parvum, Strongyloides stercoralis, Trypanosoma cruzi, Theileria ovis*, and *Echinococcus granulosus* simultaneously.

### 3.7 Validation of the TaqMan probe-based pentaplex qPCR

A comparative experiment was conducted on 16 diarrhea samples using both TaqMan probe-based pentaplex qPCR and PCR. Table 6 displayed that the results of TaqMan probe-based pentaplex qPCR were consistent with those of PCR, indicating its effectiveness in simultaneously detecting *Cryptosporidium parvum, Strongyloides stercoralis, Trypanosoma cruzi, Theileria ovis*, and *Echinococcus granulosus*.

**Table 6:** The detection results of sixteen samples of diarrhea using the TaqMan probe-based pentaplex qPCR and the PCR.

Item	Pathogen	S1	S2	S3	S4	S5	S6	S7	S8	S9	S10	S11	S12	S13	S14	S15	S16
TaqMan probe-based pentaplex qPCR	*Cryptosporidium parvum*	–	–	–	–	–	–	+	+	+	+	+	+	+	+	+	+
	*Strongyloides stercoralis*	+	+	+	+	+	+	–	–	–	–	–	–	–	–	–	–
	*Trypanosoma cruzi*	–	–	–	–	–	–	–	–	–	–	–	–	–	–	–	–
	*Theileria ovis*	–	–	–	–	–	–	–	–	–	–	–	–	–	–	–	–
	*Echinococcus granulosus*	–	–	–	–	–	–	–	–	–	–	–	–	–	–	–	–
PCR	*Cryptosporidium parvum*	–	–	–	–	–	–	+	+	+	+	+	+	+	+	+	+
	*Strongyloides stercoralis*	+	+	+	+	+	+	–	–	–	–	–	–	–	–	–	–
	*Trypanosoma cruzi*	–	–	–	–	–	–	–	–	–	–	–	–	–	–	–	–
	*Theileria ovis*	–	–	–	–	–	–	–	–	–	–	–	–	–	–	–	–
	*Echinococcus granulosus*	–	–	–	–	–	–	–	–	–	–	–	–	–	–	–	–

### 3.8 Clinical performance of the TaqMan probe-based pentaplex qPCR

**Figure 5:**
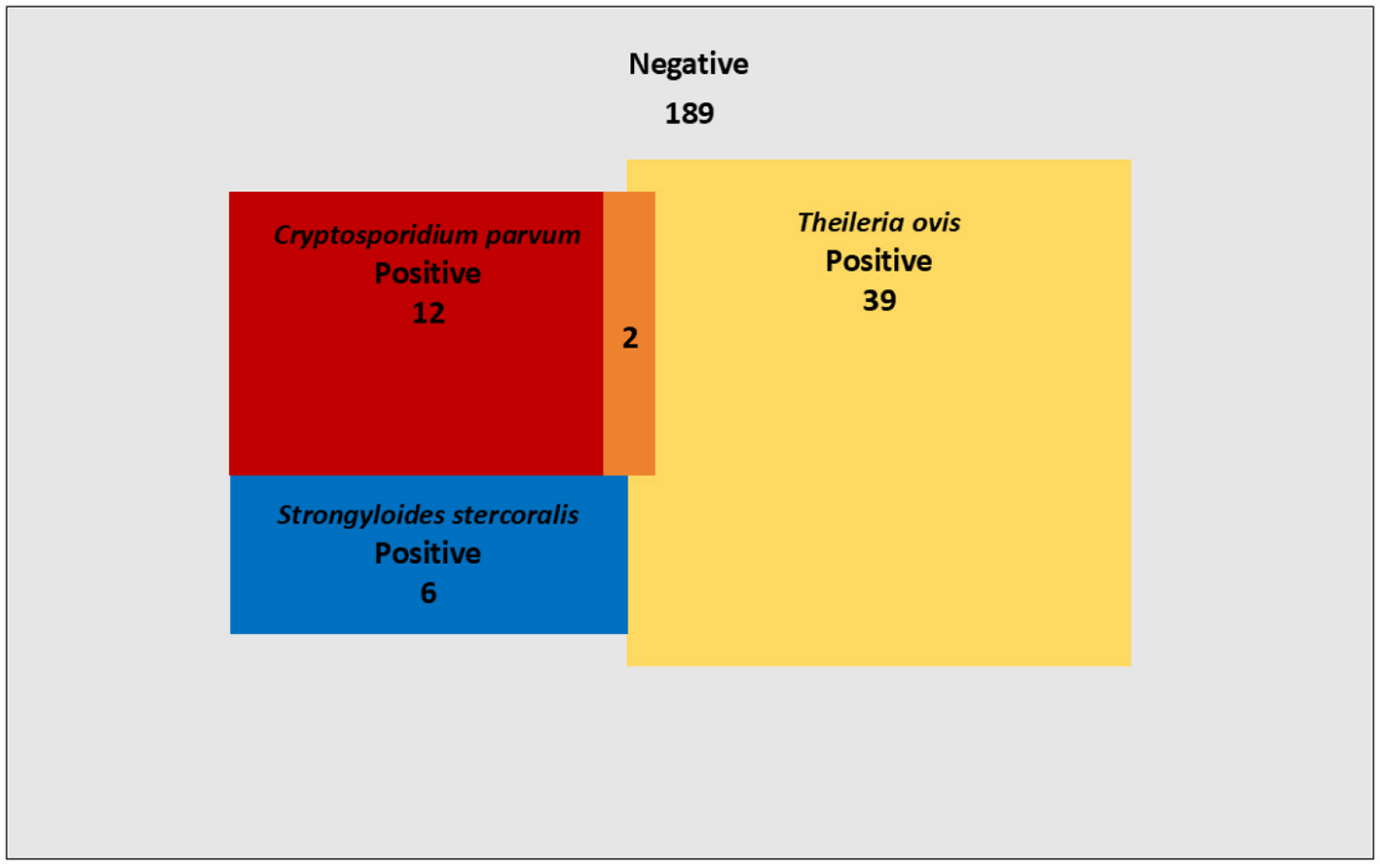
The detection results of clinical samples using the TaqMan probe-based pentaplex qPCR.

The TaqMan probe-based pentaplex qPCR was used to analyze 246 clinical samples. Figure 5 showed that the individual infection rates of *Cryptosporidium parvum, Strongyloides stercoralis*, and *Theileria ovis* were 4.88% (12/246), 2.44% (6/246), and 15.85% (39/246), respectively. The mixed infection rates were 0.81% (2/246) for *Cryptosporidium parvum*/*Theileria ovis*. The results demonstrated that the TaqMan probe-based pentaplex qPCR had an excellent ability to detect coinfections.

## 4 Discussion

Distinguishing parasitic pathogens remains a challenging task. For cases with negative results from microscopic examination, traditional diagnostic methods seem inadequate. There is an urgent need for innovative diagnostic methods to avoid misdiagnosis and inappropriate treatment. Our TaqMan probe-based pentaplex qPCR directly fills this gap. It can simultaneously detect and distinguish five clinically important pathogens (*Cryptosporidium parvum, Strongyloides stercoralis, Trypanosoma cruzi, Theileria ovis*, and *Echinococcus granulosus*) in a single sample. To our knowledge, this is the first report of using a TaqMan probe-based pentaplex qPCR to rapidly detect and differentially identify *Cryptosporidium parvum, Strongyloides stercoralis, Trypanosoma cruzi, Theileria ovis*, and *Echinococcus granulosus* in clinical samples.

Compared with previous technologies (Tako et al., 2023; Ali et al., 2023; Mota et al., 2007; Nangru et al., 2022; Zarei et al., 2019; Li et al., 2011; Yildiz et al., 2024; Gholami Koohestan et al., 2024), our TaqMan probe-based pentaplex qPCR has the following characteristics:

According to this study, the *gp60* gene, *COX1* gene, *SAT* gene, *SSU rRNA* gene and *ND1* gene were selected as the detection genes for the prevalent strains of *Cryptosporidium parvum, Strongyloides stercoralis, Trypanosoma cruzi, Theileria ovis*, and *Echinococcus granulosus*. After multiple gene sequence comparisons, highly conserved regions were selected from the *gp60, COX1, SAT, SSU rRNA* and *ND1* genes to design primers and probes, while regions with extremely low homology and numerous mutation sites were excluded. However, since the purpose of this study is to simultaneously detect these five pathogen targets in the same detection system (that is, using five fluorescence channels), if the detection system for each target is not applicable, then the Cq values of each detection will differ greatly, and/or there is mutual interference between each primer and probe, and fluorescence inhibition will affect the detection rate during the detection process. In view of this, through innovative efforts, we further designed and determined primer and probe combinations with similar amplification efficiency for detecting the nucleic acids of these five pathogens. The detection targets were short and highly conserved sequences of the five objectives. At the same time, in the TaqMan probe-based pentaplex qPCR amplification process, the ratio of primers to fluorescent probes in the detection system was adjusted. Moreover, the amplification efficiency of the nucleic acids of these different pathogens remained consistent.

When there is a mixed infection of multiple pathogens, determining the infection status of these multiple pathogens solely through a single qPCR method would be extremely time-consuming, laborious and costly. The TaqMan probe-based pentaplex qPCR provides a detection method that can simultaneously and rapidly detect *Cryptosporidium parvum, Strongyloides stercoralis, Trypanosoma cruzi, Theileria ovis*, and *Echinococcus granulosus* in the same detection system. By using specific probes labeled with five different fluorescent emission groups to target the *gp60* gene of *Cryptosporidium parvum, COX1* gene of *Strongyloides stercoralis, SAT* gene of *Trypanosoma cruzi, SSU rRNA* gene of *Theileria ovis* and *ND1* gene of *Echinococcus granulosus*, it is possible to simultaneously determine the mixed infection of multiple pathogens in a single reaction using the TaqMan probe-based pentaplex qPCR assay. It not only saves time but also reduces labor intensity. Moreover, it can significantly lower the detection cost.

In our TaqMan probe-based pentaplex qPCR, five pairs of primers-probe combinations can be mixed into a single tube system, enabling simultaneous detection of these five target pathogens in a single reaction. It is worth noting that this detection method has excellent specificity and no cross-reactivity with other non-target pathogens (including *Toxoplasma gondii, Giardia lamblia, Balantidium coli, Aspiculuris tetraptera, Syphacia obvelata*, and *Syphacia muris*). This ensures the accuracy of the detection. On the other hand, the coefficient of variation for both intra group and inter group is less than 3%, indicating that this detection method has excellent repeatability (intra group) and reproducibility (inter group). Moreover, these five targets do not interfere with each other, regardless of their concentration being high or low.

This study developed a novel TaqMan probe-based pentaplex qPCR for the rapid and simultaneous detection of *Cryptosporidium parvum, Stongyloides stercoralis, Trypanosoma cruzi, Theileria ovis*, and *Echinococcus granulosus*. This detection method has the characteristics of qualitative, quantitative, and high sensitivity (the LLOQ was 10 copies per reaction. The LOD for detecting *Cryptosporidium parvum, Strongyloides stercoralis, Trypanosoma cruzi, Theileria ovis*, and *Echinococcus granulosus* was 1.7, 1.2, 1.5, 1.4 and 1.4 copies per reaction, respectively). In our optimal system, the TaqMan probe-based pentaplex qPCR can rapidly detect and differentially identify *Cryptosporidium parvum, Stongyloides stercoralis, Trypanosoma cruzi, Theileria ovis*, and *Echinococcus granulosus* with eight logs of dynamic range, and has high analytical specificity and accuracy. Its simplified design shortens the processing time from sample to result, requiring only 83 minutes, and reduces the risk of cross-contamination. It is superior to other related reports (Higgins et al., 2001; Riahi et al., 2020).

The clinical detection performance of the TaqMan probe-based pentaplex qPCR was evaluated in clinical samples to verify its practicality and effectiveness. The results showed that 4.88%, 2.44%, and 15.85% of the samples were detected positive for *Cryptosporidium parvum, Strongyloides stercoralis*, and *Theileria ovis*, respectively. In addition, there are cases of mixed infection with two or more pathogens, which may exacerbate immunosuppression and inflammatory responses, thereby increasing the likelihood of secondary infections by other pathogens and further accelerating the progression of these diseases (Li et al., 2022; Garcia-Rejon et al., 2021). In this study, among the clinical samples, using the TaqMan probe-based pentaplex qPCR, two cases of double-positive samples for *Cryptosporidium parvum* and *Theileria ovis* were identified. This indicates that *Cryptosporidium parvum* and *Theileria ovis* are still prevalent in China, which is consistent with the results of other reports (Mi et al., 2018; Chen et al., 2022; Wang et al., 2021; Zhao et al., 2020; Wang et al., 2023). Among these 189 samples, the absence of the target pathogen detection might be due to non-infection factors or other non-target pathogens. Therefore, clinical practice must comprehensively consider various factors and take effective measures to prevent and control related diseases.

However, our experiments have some limitations. Firstly, although TaqMan probes and pentaplex qPCR primers were designed to target the highly conserved regions of *Cryptosporidium parvum* gp60, *Strongyloides stercoralis COX1, Trypanosoma cruzi SAT, Theileria ovis SSU rRNA*, and *Echinococcus granulosus ND1*, the possibility of gene mutations, deletions, and genetic recombination over time could lead to the appearance of off-target primers and probes, thereby further resulting in false negative results. Secondly, the sample size is relatively small. The number of clinical samples examined in the study was insufficient, which limited its further application in large-scale research. Additionally, in this study, the TaqMan probe-based pentaplex qPCR was only compared with the PCR method, but not with the antigen detection method.

In future research, we will collect more clinical samples and employ multiple detection methods for comparison, in order to provide more compelling evidence to demonstrate the application value of this method in large-scale clinical tests. Further development and research should focus on verifying the TaqMan probe-based pentaplex qPCR in larger-scale and more diverse populations to ensure its applicability in different populations and clinical manifestations. Expanding the research scope will help determine the reliability and adaptability of the TaqMan probe-based pentaplex qPCR, enabling its application in different geographical environments.

## 5 Conclusion

In conclusion, this study developed a TaqMan probe-based pentaplex qPCR for the detection of five clinically important pathogens (*Cryptosporidium parvum, Strongyloides stercoralis, Trypanosoma cruzi, Theileria ovis*, and *Echinococcus granulosus*) in clinical samples for the first time. This method has strong specificity and high sensitivity, providing a rapid and accurate means for the diagnosis of parasitic infections. The TaqMan probe-based pentaplex qPCR has the characteristics of high sensitivity, strong specificity, accurate qualitative determination, precise quantitative measurement, excellent repeatability and reproducibility, high throughput, short time consumption, low labor consumption, and cost-effectiveness. This method is applicable to the differential diagnosis of mixed clinical infections, thereby facilitating early diagnosis and treatment. This may play a significant role in preventing and controlling the transmission of infectious diseases. Its stable analytical performance, high diagnostic accuracy, efficient process, and cost-effectiveness make it a promising tool for the rapid detection of parasitic pathogen infections in resource-poor areas. This is of great significance for safeguarding human and public health.

## Data Availability

The original contributions presented in the study are included in the article/supplementary material, further inquiries can be directed to the corresponding authors.
